# Transverse Sinus Stenosis as an Underdiagnosed Cause of Chronic Headache: A Case Report

**DOI:** 10.7759/cureus.67206

**Published:** 2024-08-19

**Authors:** Marina Handal, RaeAnn Tourangeau-Young, Alejandro Biglione

**Affiliations:** 1 Medicine, Dr. Kiran C. Patel College of Osteopathic Medicine, Nova Southeastern University, Fort Lauderdale, USA; 2 Internal Medicine, Wellington Regional Medical Center, Wellington, USA

**Keywords:** a case report, transverse sinus stenting, transverse sinus stenosis, migraine headaches, chronic daily headache

## Abstract

Transverse sinus stenosis (TSS) is an abnormality in the cerebral venous system in which the narrowing of the transverse sinus of the brain leads to obstructed cerebral venous outflow. It is an infrequent, incidental radiological finding. However, it is not uncommon among patients with chronic headaches of unclear cause, particularly those that remain unexplained after initial evaluation or those that are refractory to medical treatment. Its diagnosis frequently eludes the initial workup, and a high degree of suspicion should be maintained since its identification can lead to potentially curative treatment. This report describes the case of a 36-year-old female with a history of chronic headache who was found to have TSS. This paper discusses its etiology, pathophysiology, clinical presentation, radiological findings, and management.

## Introduction

Headache is one of the most common reasons for patients to seek medical attention. The prevalence of headaches is approximately 40% of the adult population worldwide, making it one of the most frequent presenting complaints diagnosed by physicians [1]. Headache disorders result in significant disability, often affecting patient quality of life and productivity. Published by the International Headache Society (IHS), the International Classification of Headache Disorders (ICHD) is a universal diagnostic tool used for the clinical assessment, classification, and management of headaches. It outlines three main types of headaches: primary headaches, secondary headaches, and painful cranial neuropathies such as trigeminal neuralgia [2]. Accounting for 90% of headaches, primary disorders include migraine, tension-type headache (TTH), and cluster headaches. The most frequent type of primary headache in the outpatient setting is TTHs. TTHs are bilateral and non-pulsatile in quality and result in dull, aching pain along the forehead and temples. In contrast to migraine headaches, TTHs are not accompanied by photophobia, phonophobia, or aura [3].

Migraine headaches occur in 12%-15% of the population and are described as unilateral, pulsating, and episodic. They may be associated with photophobia and nausea. Migraines are occasionally preceded by an aura, which is a reversible visual or somatosensory phenomenon consisting of paresthesias, tinnitus, or hearing and vision loss. Cluster headaches are seen in 0.2% of the population and are characterized by excruciating, unilateral pain along the periorbital and temporal regions of the face, commonly involving the first division of the trigeminal nerve. Patients may experience conjunctival injection, miosis, eyelid edema, diaphoresis, Horner syndrome, and a sensation of fullness in the ear [4]. In patients with high-risk features that are not compatible with primary headache, secondary headache disorders must be investigated. Secondary headaches are usually associated with “red flag” features, including onset over the age of 50 years, severe and sudden presentation, altered mental status, seizures, cognitive impairment, focal deficits, stiff neck, and papilledema. Headaches associated with exertion, head trauma, drugs, and toxins are also of clinical importance [5]. A headache that occurs more than 15 times a month for three months is described as a chronic headache [6].

An unusual cause of chronic headache is transverse sinus stenosis (TSS). TSS is an abnormality of the cerebral venous system that involves the narrowing of the transverse sinus of the brain resulting in obstructed venous outflow. TSS is an uncommon radiological finding, with a prevalence of 7% among patients with chronic headache [7]. Its prevalence is much higher among patients who remain undiagnosed after initial diagnostic studies and those who are unresponsive to medical treatment. It is important to maintain a high degree of suspicion because cerebral angiography with venous phase may be required for timely diagnosis and treatment [8,9]. This report describes the case of a 36-year-old patient with chronic headaches with unclear cause refractory to medical treatment who was eventually found to have stenosis of the right transverse sinus and was successfully treated with transverse sinus stenting.

## Case presentation

The patient was a 36-year-old Caucasian female with chronic headaches worsening over the last six months. She described her daily headaches as dull, generalized, and moderate-intensity pain level. The headaches were exacerbated by physical activity and cough. She denied radiation, nausea, aura, or focal neurological symptoms. She had a past medical history of attention deficit hyperactivity disorder. Her medications included dextroamphetamine-amphetamine extended release 20 mg daily.

The patient was evaluated with multimodal brain imaging in the outpatient setting. A CT of the head with and without contrast was normal. An MRI of the brain with and without gadolinium was normal. A magnetic resonance venography (MRV) of the brain was also normal. Due to persistent symptoms, a cerebral angiogram was recommended. The patient had an elective cerebral angiogram with venogram and 3D reconstruction imaging. Her vital signs showed a temperature of 37.3°C, a heart rate of 65 beats per minute (bpm), a respiratory rate (RR) of 20 breaths per minute, a blood pressure of 120/74 mmHg, and an oxygen saturation of 99%. Her BMI was 22.15 kg/m^2^.

On physical examination, her neck was supple without palpable masses. Her chest was clear to auscultation, and her heart showed normal S1 and S2 with regular rhythm and without murmurs. The abdomen was normal to inspection, non-tender to palpation, without palpable organomegaly or masses, and with normal bowel sounds. The lower extremities showed no signs of pedal edema. Her neurological examination did not show any abnormalities in her mental status, cranial nerves, motor system, sensory system, coordination, gait, or reflexes. The fundoscopy was normal. The patient’s laboratory results are displayed in Table 1 and Table 2.

**Table 1:** General chemistry

Laboratory Parameters	Patient Value	Reference Value
Glucose level	96 mg/dL	74-106 mg/dL
Sodium	139 mmol/L	135-148 mmol/L
Potassium	3.9 mmol/L	3.6-5.2 mmol/L
Chloride	108 mmol/L	95-110 mmol/L
Carbon dioxide	25.0 mEq/L	21.0-32.0 mEq/L
Anion gap	9.9 mmol/L	5.0-15.0 mmol/L
Blood urea nitrogen	9 mg/dL	7-18 mg/dL
Creatinine	0.67 mg/dL	0.55-1.02 mg/dL
Calcium	8.5 mg/dL	8.5-10.1 mg/dL
Albumin level	4.1 g/dL	3.4-5.0 g/dL
Total bilirubin	1.0 mg/dL	0.0-1.0 mg/dL
Alkaline phosphatase	68 IU/L	45-117 IU/L
Aspartate aminotransferase	16 IU/L	15-37 IU/L
Alanine aminotransferase	19 IU/dL	12-78 IU/L
Magnesium level	2.2 mg/dL	1.8-2.4 mg/dL
Phosphorus	3.3 mg/dL	2.5-4.9 mg/dL

**Table 2:** General hematology

Laboratory Parameters	Patient Value	Reference Value
White blood cells	4.40 × 10^3 ^mcL	4.50-10.50 × 10^3 ^mcL
Red blood cells	4.70 × 10^6^ mcL	4.20-5.50 × 10^6^ mcL
Hemoglobin	14.2 g/dL	12.0-16.0 g/dL
Hematocrit	41.0%	37.0%-47.0%
Mean corpuscular volume	87.2 fL	81.0-96.0 fL
Mean corpuscular hemoglobin	30.2 pg	27.0-34.0 pg
Mean corpuscular hemoglobin concentration	34.6 g/dL	32.0-36.0 g/dL
Platelets	288 × 10^3 ^mcL	150-450 × 10^3 ^mcL
Mean platelet volume	10.0 fL	6.9-10.5 fL

**Figure 1:**
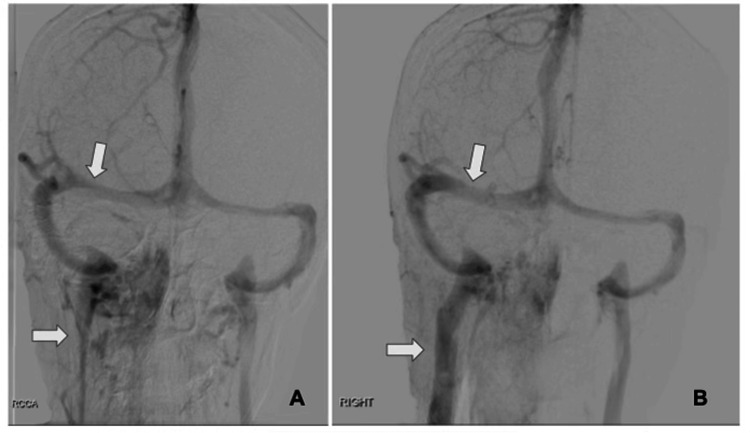
(A) Diagnostic cerebral angiogram with venogram and 3D reconstruction imaging showing moderate-to-severe stenosis in the right transverse venous sinus and the right internal jugular vein. (B) Following a cerebral angiogram and successful stenting of the right transverse sinus and the right internal jugular vein stenosis.

Her cerebral angiogram revealed moderate-to-severe stenosis of the right transverse sinus and the right internal jugular vein (IJV) (Figure 1A). Due to this finding, she was admitted to the hospital and scheduled to undergo right IJV and right transverse venous sinus stenting the next day. Daily aspirin 81 mg and clopidogrel 75 mg were started. Cerebral angiogram and stenting of the right transverse sinus and the right IJV were successfully performed the next day as planned (Figure 1B). Her postoperative course was uneventful. She was discharged from the hospital on daily aspirin and clopidogrel. In her follow-up visit, she reported the resolution of her headaches.

## Discussion

The prevalence of TSS among patients with chronic headache is approximately 7%, regardless of gender. TSS is a rare cause of headaches and is more prevalent in patients who are not diagnosed after standard brain imaging studies including the CT or MRI of the brain [7]. TSS is a condition affecting the cerebral venous system, characterized by the narrowing of the transverse sinus of the brain resulting in the restriction of venous outflow. TSS may be caused by extrinsic compression or intrinsic obstruction of the cerebral venous sinuses. Extrinsic compression by the adjacent brain parenchyma can lead to the compression of the sinuses against the skull [8]. Because veins are composed of elastic tissue and smooth muscle, they are easily flattened and therefore collapsible when subject to compression. Intrinsic obstruction can be caused by protrusions of enlarged arachnoid granulations, chronic thrombosis, or stenosis by fibrous septa. Cerebral venous sinuses allow for the draining of blood from the cranial cavity. Sinus vein obstruction alters venous dynamics causing outflow restriction of the CSF drainage that produces intracranial hypertension (IH) [8,9]. This flow abnormality may result in poor venous sinus drainage with congestion and could predispose patients to dural sinus thrombosis [10].

TSS can be diagnosed by noninvasive radiographic modalities including CT venography (CTV) and contrast-enhanced magnetic resonance venography (MRV). While studies have yet to define definitive radiographic criteria for TSS, stenosis that entails >50% reduction in vessel diameter is considered diagnostic. On the CTV and MRI-MRV of the brain, TSS appears as a collapse of the transverse venous sinus, with a full absence of a definable sinus in one or more sections of the brain or a herniation of the contour of the brain into the sinus [7,9]. MRV is a better diagnostic tool compared with CTV and detects approximately 90% of cases [11]. The diagnosis of TSS may still remain elusive after the CTV and MRI-MRV of the brain. Cerebral angiography with venous phase may be required for diagnostic confirmation [12,13]. On cerebral angiography, TSS due to extrinsic compression appears as a gradually narrowing tapered stenosis; in contrast, intrinsic stenosis due to arachnoid granulations or septa can be visualized as discrete, intraluminal filling defects [14]. The patient in this case also revealed the stenosis of the ipsilateral internal jugular vein (IJV) concomitantly with the stenosis of the right transverse sinus. This combination makes this case even more unusual as impaired flow in the ipsilateral IJV could accentuate the symptomatology derived from the TSS.

It is critical to maintain a high index of suspicion and consider TSS in the differential diagnosis of patients with chronic headaches. Venous sinus stenting is an effective and safe intervention for patients experiencing chronic headaches unresponsive to conservative medical treatment, including the chronic use of opioid analgesics that are associated with the devastating risks of dependency. Transverse sinus stenting alleviates cerebral venous obstruction by improving CSF drainage, therefore lowering intracranial and venous sinus pressures. Approximately 80% of patients experience the improvement or complete resolution of their headaches following stenting [14]. The possible complications of stent placement include intracerebral bleeding due to guidewire perforation, acute thrombosis or stenosis of the stent leading to ischemic or hemorrhagic stroke, subdural hematomas, transient hearing loss, or ipsilateral frontal headaches due to dural stretching. However, such risks are unusual, occurring in no more than 3% of cases [14,15]. Therefore, transverse venous sinus stenting is a safe intervention recommended for definitive management in patients experiencing chronic headaches due to TSS [16,17].

## Conclusions

TSS is an underdiagnosed cause of chronic headache. It must be considered as part of the comprehensive evaluation of patients with chronic headaches, particularly in those cases that remain unexplained and are refractory to medical treatment. It may elude initial imaging tests including the CT and MRI of the brain. The MRV of the brain offers the highest diagnostic yield. Cerebral angiography with venous phase is the gold standard for its diagnosis. The stenting of the transverse sinus is a safe procedure that may result in the resolution of chronic headaches associated with TSS.
